# The Prognostic Value of Epigenetic Silencing of *p16* Gene in NSCLC Patients: A Systematic Review and Meta-Analysis

**DOI:** 10.1371/journal.pone.0054970

**Published:** 2013-01-25

**Authors:** Zhang Lou-Qian, Yin Rong, Li Ming, Yang Xin, Jiang Feng, Xu Lin

**Affiliations:** Department of Thoracic Surgery, Jiangsu Cancer Hospital, Affiliated Cancer Hospital of Nanjing Medical University, Nanjing, China; Rush University Medical Center, United States of America

## Abstract

**Background:**

The prognostic significance of *p16* promoter hypermethylation in patients with non-small cell lung cancer (NSCLC) is still controversial. This analysis presents pooled estimates of the association to better elucidate whether *p16* methylation has a prognostic role in NSCLC.

**Methods:**

Relevant studies were identified by searching PubMed, Embase and Web of Science databases until June 2012. The association of *p16* methylation with both overall survival (OS) and disease-free survival (DFS) was preformed. Studies were pooled and summary hazard ratios (HR) were calculated. Subgroup analyses, sensitivity analysis and publication bias were also conducted.

**Results:**

A total of 18 studies containing 2432 patients met the inclusion criteria and had sufficient survival data for quantitative aggregation. The results showed that *p16* methylation was an indicator of poor prognosis in NSCLC. The HR was 1.36 (95% CI: 1.08–1.73, I^2^ = 56.7%) and 1.68 (95% CI: 1.12–2.52, I^2^ = 38.7%) for OS and DFS, respectively. Subgroup analyses were carried out. The HRs of fresh and paraffin tissue were 1.50 (95% CI: 1.11–2.01) and 1.10 (95% CI: 0.77–1.57). The pooled HR was 1.40 (95% CI: 1.02–1.92) for methylation-specific PCR (MSP) and 1.26 (95% CI: 0.87–1.82) for quantitative MSP (Q-MSP). The combined HR of the 16 studies reporting NSCLC as a whole indicated that patients with *p16* hypermethylation had poor prognosis. No significant association was found when adenocarcinoma subtype pooled. When seven studies on DFS were aggregated, the HR was 1.68 (95% CI: 1.12–2.52) without significant heterogeneity. Moreover, no obvious publication bias was detected on both OS and DFS.

**Conclusion:**

The meta-analysis findings support the hypothesis that *p16* methylation is associated with OS and DFS in NSCLC patients. Large well-designed prospective studies are now needed to confirm the clinical utility of *p16* methylation as an independent prognostic marker.

## Introduction

With more than 1.6 million cases diagnosed annually, lung cancer is the leading cause of cancer-related deaths in men and the second leading cause of cancer deaths in women worldwide [1]. Given the clinical burden of lung cancer, with non–small cell lung cancer (NSCLC) accounting for approximately 85% of all cases, it has become a major health problem, worldwide. Despite major advances in cancer treatment in the past two decades, the prognosis of patients with lung cancer has improved only minimally, the overall 5-year survival rate for NSCLC (all stages combined) is roughly 15% (http://seer.cancer.gov/statfacts/html/lungb.html).

Although TNM stage is the most significant clinical parameter to be considered on cancer prognosis, the variability of survival within staging groups requires additional parameters affecting outcome, independent of tumor stage. Since multiple gene and gene-related alterations contribute to NSCLC development and progression. Therefore, determination of the biological behavior and identification of prognostic biomarkers is important for the early detection of relapse, as well as for stratification of patients before enrollment onto their treatment regimens. There is increasing evidence that epigenetic alterations, particularly inactivation of tumor suppressor genes or tumor-related genes through promoter hypermethylation, play a dominant role in various cancers including lung cancer [2], [3]. Promoter CpG island methylation is the most widely studied and best characterized epigenetic alteration in NSCLC, providing some of the most promising markers for early detection and prediction of prognosis or treatment response in NSCLC (see review article [4]).

The *p16* ^INK4A^ gene is known as a tumor suppressor gene, which functions as negative regulator of the cell cycle progression through its inhibition of cdk4/6 and subsequent blockage of the cyclin-dependent phosphorylation of the Rb [5]. Promoter silencing of *p16* ^INK4A^ through methylation lead to loss of control of the restriction point in the G1 phase of the cell cycle and favor cellular transformation [6], [7], [8]. Abnormal *p16* promoter hypermethylation has been found in several types of tumor, and it is inactivated in 40% to 70% of NSCLC patients [8]. The contribution of *p16* deregulation through methylation to the carcinogenic process has been extensively studied. Moreover, bulks of observational studies evaluating its prognostic value in NSCLC have been carried out in past ten years [9], [10], [11], [12], [13], [14], [15], [16], [17], [18], [19], [20], [21], [22].

The prognostic role of *p16* methylation in NSCLC has been investigated over the past decade, with conflicting results from different literatures. Some studies concluded *p16* hypermethylation was an independent prognostic factor for dismal outcome. Some other studies did not identify this association. In order to clarify this question, we sought to conduct a systematic review and meta-analysis to estimate the prognostic importance of *p16* methylation status for overall survival (OS) and disease-free survival (DFS) in patients with NSCLC.

## Materials and Methods

### Literature Search Strategy

We performed a systematic search of the relevant literature using PubMed, Web of Science and Embase databases up to June 2012 without language restrictions. We used the following search terms and their combinations: “NSCLC,” “non-small-cell lung cancer,” “lung cancer,” “*p16*,” “*p16* ^INK4a^,” and “methylation.” Moreover, the references in all retrieved articles were screened to identify additional articles that were not identified in the initial literature search described above.

### Selection Criteria

To be included in this meta-analysis, the studies should meet the following criteria: 1. full text publication with details of methods available; 2. Contained data on assessment of *p16* methylation status; 3. Contained outcome data for NSCLC patients according to *p16* methylation status (outcomes were overall survival and/or disease-free survival); 4. hazard ratio (HR) for OS or DFS according to *p16* status either had to be reported or could be calculated from the sufficient data provided in the original article; 5. to avoid duplicated publications, we included the most recent report or the most complete one.

### Data Extraction

The following information was drawn from each eligible study: first author, year of publication, number of patients participated, number of patients with *p16* methylation status, TNM stage, histology, testing material, detecting methods, and data linking *p16* methylation status and clinical outcome (OS and DFS). The whole process of data extraction was performed independently by two authors, and the disparities were solved by discussion.

### Statistical Methods

The hazard ratio (HR) was abstracted or calculated to quantitatively evaluate the association between *p16* methylation status and NSCLC prognosis. The summary HR for overall survival and disease-free survival were evaluated by calculating pooled Cox proportional hazard ratios and 95% confidence intervals (CI) as relevant effect measures using previously published methods. When related data were not available directly from the studies, we calculated the corresponding HR and its 95%CI using the method described by Tierney et al [23]. We then investigated the between-study heterogeneity by using the Cochran’s Q test, using a significance level of p value less than 0.1. The statistic I^2^ was also used to quantitatively evaluate the heterogeneity [24]. If I^2^ greater than 50% is considered a measure of severe heterogeneity, then the random-effects model was adopted to calculate HR according to the DerSimonian–Laird method [25]. Otherwise, the fixed-effects model (Mantel–Haenszel method) was used [26]. The assessment of sources of heterogeneity was undertaken by meta-regression analysis and subgroup analyses. One-way sensitivity analysis was performed to assess the stability of the results, namely, a single study in the meta-analysis was deleted each time to reflect the influence of the individual data set to the pooled HR [27]. We also used inverted funnel plots and the Egger’s test to examine the effect of publication bias (linear regression analysis) [28]. All p values were 2-sided and less than 0.05 was considered as significance. All analyses were carried out on STATA 11.0 software platform (Stata Corporation, College Station, TX).

## Results

### Selection and Characteristics of Studies

By the initial literature search, 145 records were identified regarding the association of *p16* methylation status and NSCLC survival; 111 studies were excluded after screening the titles or abstracts because they were either review articles, abstracts, no on human being, duplicate publication, or studies irrelevant to the current theme (mainly on cancer early diagnosis). Thirty-four relevant studies were selected for detailed evaluation. Nine were excluded for the systematic review after full assessment (7 were lacking relevant survival data, another 2 were duplications). Of the 25 remained studies, eighteen studies were eligible for the meta-analysis [9], [12], [13], [14], [15], [18], [19], [22], [29], [30], [31], [32], [33], [34], [35], [36], [37], [38], 7 provided insufficient data for performing a quantitative aggregation [11], [17], [20], [39], [40], [41], [42].

The main features of the 25 studies eligible for the systematic review are shown in Table 1. The number of patients included across all studies varied from 43 to 335. Among the 25 studies, 13 reported that *p16* hypermethylation was an unfavorable indicator of survival; the other 12 studies didn’t found this association. There were 18 studies containing 2432 patients had sufficient survival data for quantitative aggregation, among which, 14 studies used methylation-specific PCR (MSP) to assess gene methylation status, and 4 used quantitative MSP (Q-MSP) to determine its methylation status. Seventeen studies dealt with NSCLC of all histological subtypes, one with adenocarcinoma only [32]. Moreover, five trials reported the results of adenocarcinoma in stratified analysis [29], [30], [32], [33], [37]. Twelve trials detected *p16* methylation by using fresh frozen tissue and 5 using formalin-fixed paraffin-embedded (FFPE) specimen, only one using peripheral blood as sample [14].

**Table 1:** The main characteristics and results of eligible studies evaluating p16 hypermethylation and NSCLC patients’ survival.

First author	Year	Country	Number	Method	M	U	Stage	Material	Histology	Estimate	Results	OS	DFS
												HR (95%CI)	HR (95%CI)
Duk-Hwan Kim [22]	2001	USA	185	MSP	51	134	I–IV	fresh tissue	NSCLC	Cox	S	4.72(1.02–21.85 )	NA
Niklinska [42]	2001	Poland	52	MSP	16	36	I–II	fresh tissue	NSCLC	Cox	S	NA	NA
Fu [41]	2003	China	64	MSP	36	28	I–III	fresh tissue	NSCLC	NA	S	NA	NA
Harden [20]	2003	USA	90	MSP	15	75	I	fresh tissue	NSCLC	NA	NS	NA	NA
Maruyama [19]	2004	USA	124	MSP	25	99	I–IV	fresh tissue	NSCLC	Cox	NS	0.81(0.43–1.52)	NA
Shimamoto [40]	2004	Japan	45	MSP	17	28	I–III	fresh tissue	NSCLC	K–M	S	NA	NA
Toyooka [29]	2004	Japan	351	MSP	86	265	I–III	fresh tissue	NSCLC	Cox	S	1.82(1.10–3.00)	NA
			105					fresh tissue	ADC		S	2.57(1.15–5.76)	NA
Wang [18]	2004	USA	119	MSP	58	61	I–IIIA	fresh tissue	NSCLC	K–M	S	2.26(1.26–4.06)	NA
			49	MSP	24	25	IIIA	fresh tissue	NSCLC	K–M	S	2.72(1.14–5.13)	1.46(1.14–6.81)
			70	MSP	37	33	I–II	fresh tissue	NSCLC	K–M	S	1.69(1.07–6.98)	1.51(1.21–7.63)
Divine [17]	2005		147	MSP	63	84	I	FFPE tissue	ADC	K–M	NS	NA	NA
Young Tae Kim [30]	2005	Korea	61	MSP	41	20	I–III	fresh tissue	NSCLC	Cox	NS	0.390 (0.066–2.293)	0.509 (0.164–1.577)
			72	MSP	60	12	I–IV	fresh tissue	ADC	Cox	NS	0.176 (0.029–1.074)	NA
Safar [31]	2005	USA	105	MSP	41	64	I–IV	FFPE tissue	NSCLC	Cox	NS	0.73(0.43–1.23)	NA
Tanaka [32]	2005	Japan	57	MSP	23	34	I–III	FFPE tissue	ADC	K–M	S	1.39(1.09–4.71)	NA
Gu [33]	2006	USA	155	Q-MSP	34	121	I–III	fresh tissue	NSCLC	Cox	S	1.95(1.12–3.39)	NA
					18	59		fresh tissue	ADC	Cox	NS	1.24 (0.59–2.60)	NA
Jin Seuk Kim [38]	2006	Korea	335	MSP	117		I–IV	FFPE tissue	NSCLC	NA	NS	NA	NA
			198	MSP			I	FFPE tissue	NSCLC	Cox	S	2.67(1.21–7.64)	2.03 (1.09–6.23)
Ota [15]	2006	Japan	244	Q-MSP	87		I–IV	FFPE tissue	NSCLC	Cox	S	1.005(1.003–1.008)	NA
Sugio [34]	2006	Japan	224	MSP	49		I–IV	fresh tissue	NSCLC	log–rank	NS	2.31(0.64–9.82)	NA
Fischer [14]	2007	Germany	92	MSP	22	63	IIIB–IV	blood sample	NSCLC	log-rank	NS	1.47(0.61–3.57)	NA
Yanagawa [35]	2007	Japan	101	MSP	27	74	I–III	fresh tissue	NSCLC	Cox	NS	0.93(0.33–2.62)	NA
Brock [36]	2008	USA	187	MSP			I	FFPE tissue	NSCLC	Cox	S	NA	3.55(1.77–7.13)
Alaa [37]	2009	Japan	88	MSP	30	58	I–IV	fresh tissue	NSCLC	Cox	NS	1.4(0.6–3.2)	NA
			43	MSP	10	33	I–IV	fresh tissue	ADC	Cox	S	2.4(1.8–69.7)	NA
Yoshino [13]	2009	Japan	44	MSP	11	33	IA	fresh tissue	NSCLC	K–M	S	2.36 (1.14–9.90)	2.18 (1.56–11.2)
Bradly [39]	2010	USA	196	MSP			I–II	fresh tissue	NSCLC	NA	S	NA	NA
Buckingham [12]	2010	USA	132	Q-MSP			I–II	fresh tissue	NSCLC	K–M	NS	1.16 (0.67–2.02)	1.35 (0.74–2.46)
Sasaki [11]	2010	Japan	221	MSP	91		I–IV	fresh tissue	NSCLC	NA	NS	NA	NA
Kang [9]	2012	Korea	80	Q-MSP	9		I–III	fresh tissue	NSCLC	K–M	S	2.31 (2.10–51.72)	NA

MSP, Methylation-specific PCR; Q-MSP, quantitative methylation-specific PCR; FFPE, formalin-fixed paraffin-embedded; ADC, adenocarcinoma; S, significant; NS,not significant; NA, not available.

### Meta-analysis Results

Using the methods described above, the overall survival and/or disease-free survival of 2432 patients in 18 studies were analyzed. The main results of this meta-analysis are summarized in Table 2.

**Table 2:** The results of meta-analysis on NSCLC overall survival and p16 methylation.

	Pts number	HR (95%CI)	Heterogeneity		
			chi-squared (d.f.)	p-value	I^2^
All studies	2432	1.36 (1.08–1.73)	41.60 (d.f. = 18)	0.001	56.7%
Subgroups					
Detecting method					
MSP	1821	1.40 (1.02–1.92)	26.69 (d.f. = 14)	0.021	47.5%
Q-MSP	611	1.26 (0.87–1.82)	6.80 (d.f. = 3)	0.079	55.9%
Stage					
I	242	2.53(1.26–5.11)	0.03 (d.f. = 1)	0.865	0.0%
I–II	202	1.28(0.79–2.06)	0.46 (d.f. = 1)	0.498	0.0%
I–III	805	1.61(1.19–2.19)	4.57 (d.f. = 5)	0.470	0.0%
I–IV	1091	1.00(0.71–1.41)	11.39 (d.f. = 6)	0.077	47.3%
Histology					
NSCLC	2303	1.41(1.10–1.79)	37.28 (d.f. = 16)	0.002	57.1%
ADC	412	1.56(0.84–2.88)	9.76(d.f. = 5)	0.082	48.8%
Sample type					
Fresh tissue	1736	1.50(1.11–2.01)	20.64(d.f. = 13)	0.080	37.0%
FFPE tissue	604	1.10(0.77–1.57)	6.50(d.f. = 3)	0.090	53.8%

MSP, Methylation-specific PCR; Q-MSP, quantitative methylation-specific PCR; FFPE, formalin-fixed paraffin-embedded; ADC, adenocarcinoma; Pts, patients.

**Figure 1:**
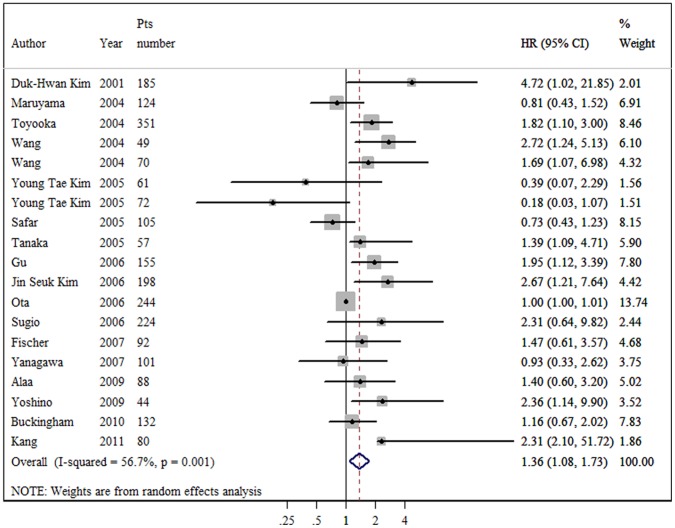
Forest plot showing the association between *p16* methylation and overall survival of NSCLC. The summary HR and 95% CIs were shown (random-effects model analysis).

Overall survival: of the 17 evaluable studies for OS, the promoter hypermethylation of *p16* was associated with a significant poor impact on NSCLC survival. However, the test for heterogeneity was highly significant (P = 0.001, I^2^ = 56.7%). Consequently, we applied a random-effect model in calculating the aggregated HR, which was 1.36 (95% CI: 1.08–1.73) (as Figure 1 shows). We also conducted subgroup analyses on sample type, detecting methods, histology, and tumor stage. When grouped according to the sample type of individual studies, the combined HRs of fresh frozen tissue and FFPE specimen were 1.50 (95% CI: 1.11–2.01) and 1.10 (95% CI: 0.77–1.57), respectively. When grouped according to the method of *p16* methylation detection used, the combined HR was 1.40 (95% CI: 1.02–1.92) for MSP and 1.26 (95% CI: 0.87–1.82) for Q-MSP. Stratified by histology, the combined HR of the 16 studies reporting NSCLC as a whole indicated that patients with *p16* hypermethylation had a risk of death 1.41 times greater than patients without *p16* hypermethylation (HR = 1.41, 95% CI: 1.10–1.79). However, no such statistical significant death risk when pooling the 6 trials of adenocarcinoma subtype (HR = 1.56, 95% CI: 0.84–2.88). We also analyzed the data according to tumor stage. For the studies focusing on stage I and I-III, the aggregation produced statistically significant HRs without significant between-study heterogeneity (2.53 and 1.61, respectively). No significant association was found in I-II and I-IV subgroups.

**Figure 2:**
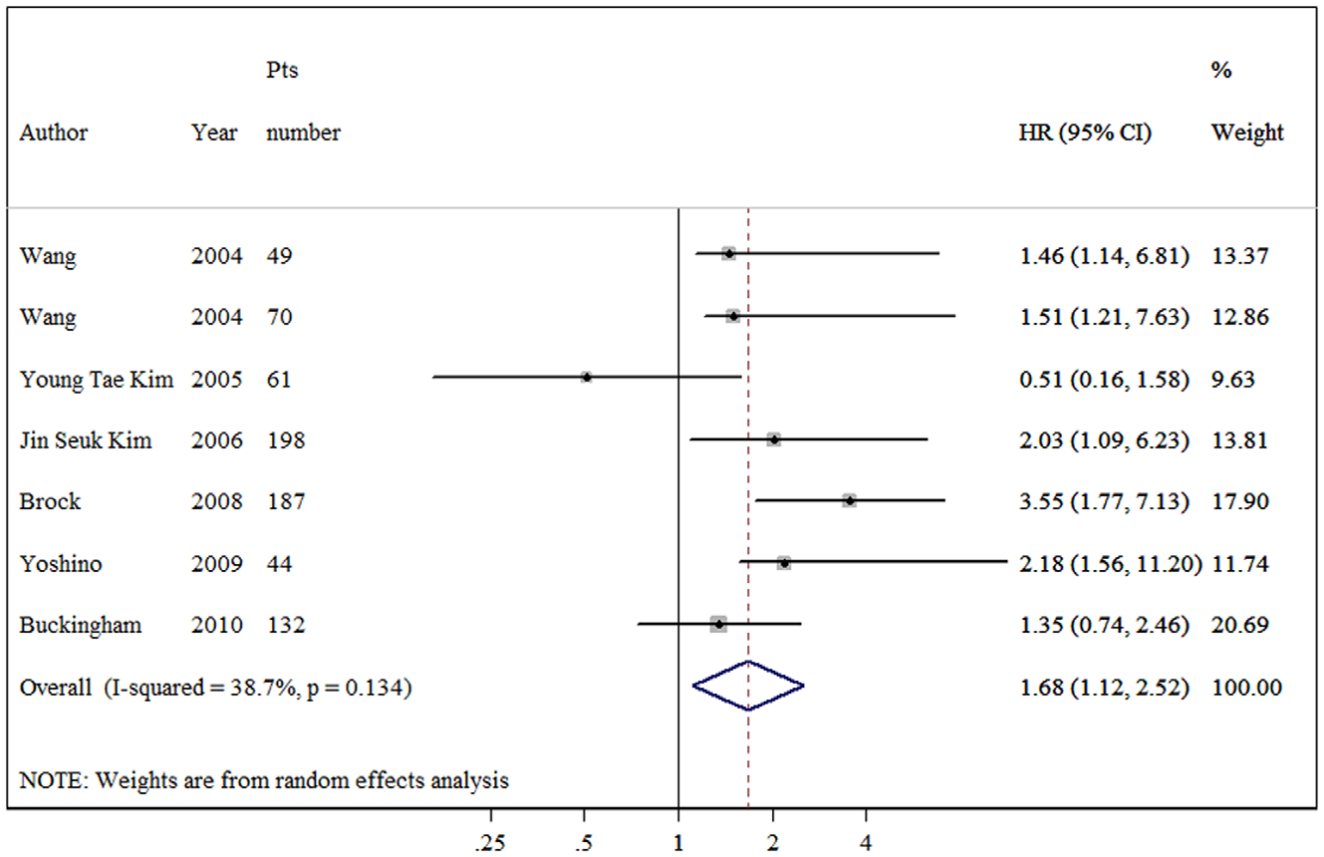
Forest plot showing the association between *p16* methylation and disease-free survival of NSCLC. The summary HR and 95% CIs were shown (random-effects model analysis).

Disease-free survival: there were seven studies with 741 patients reported the association of *p16* promoter hypermethylation and disease-free survival. NSCLC patients with *p16* promoter hypermethylation were significantly correlated with shorter DFS, and the combined HR was 1.68 (95% CI: 1.12–2.52) without significant heterogeneity (p = 0.134, as figure 2 shows). In the stratified analyses, *p16* methylation was significantly correlated with poor DFS according to MSP method, with a combined HR of 1.75 (95% CI: 1.07–2.87). When four trials using fresh tissue pooled, null association was observed (HR = 1.34, 95% CI: 0.92–1.96). The two studies with 358 cases using FFPE specimen were also combined, and the pooled HR was 2.86 (95% CI: 1.66–4.92).

### Sensitivity Analyses and Publication Bias

Sensitivity analyses were conducted by omitting one study per time in an attempt to check if individual study affected the final results of both OS and DFS. All the results were not materially altered. The visualization of the funnel plots for OS and DFS seemed asymmetrical. Egger’s test was used to provide statistical evidence for funnel plot symmetry. The results did not suggest significant evidence of publication bias: OS (Egger’s test, p = 0.05) and DFS (Egger’s test, P = 0.06).

## Discussion

No consensus has been reached regarding whether *p16* promote methylation is a prognostic marker in NSCLC patients, and it has attracted considerable attention. The present meta-analysis summarized for the first time all of the available researches on the impact of the promoter methylation of *p16* on the survival of NSCLC. The overall pooled analyses of the associations between *p16* methylation and NSCLC outcomes demonstrated negative impacts on patients with hypermethylation. As in the subgroup analyses for OS, the results suggested that *p16* methylation was a poor prognostic indicator using MSP method, whereas those using Q-MSP method combined didn’t get a similar conclusion. Individual study selected various cutoff points of the ratio for *p16* gene to dichotomize the patients into methylated and unmethylated groups might partly explain the disparity. When stratified analysis was conducted about different stages of NSCLC, the associations were also found in stages I to III, but not I-IV, with relatively larger sample pooled indicating that late-stage NSCLC patient confounded the observation of *p16* methylation on survival. About test materials, we found that the association was significant by using fresh tissue, but not paraffin block. The reliability and reproducibility of the previously developed MSP method require high-quality DNA, often from a fresh frozen specimen. In most clinical settings, although paraffin specimens are available for easy transport and storage, it might cause false negative. One study reported that MSP can provide reliable gene methylation status on only two-thirds of formalin-fixed paraffin-embedded tumor specimens compared with fresh frozen tissues [43]. When histology type was taken into account, a dismal impact on survival was observed in NSCLC, but not in adenocarcinoma which was based on the data of 6 studies. As for DFS, cases with *p16* promoter hypermethylation were significantly correlated with shorter DFS and no evidence of significant heterogeneity was found, suggesting a short recurrence. In subgroup analysis according to the different techniques used to detect *p16* methylation, an adverse impact on DFS was observed with MSP method. Interestingly, such an association with DFS was found in the studies pooled using FFPE specimens, while fresh frozen tissues were not.

The search for a potential prognostic role of *p16* methylation in NSCLC survival is based on its frequent hypermethylation in NSCLC and also on its potential interference with most pathways implicated in tumorigenesis. The role of *p16* methylation in carcinogenesis has widely been investigated by *in vitro* experiments and by *in vivo* analyses based on animal models [44], [45], [46]. Hypermethylation of CpG islands located in the promoter regions of *p16* is now firmly established as an important mechanism for gene inactivation, and the CpG island hypermethylation has been described in almost every tumor type [47]. The meta-analysis results suggested that *p16* methylation status could be used as a stratified factor for NSCLC patient survival though with slight significant associations. Increasing data suggests that DNA methylation measurements of the promoter regions of specific genes have the potential to supply additional or superior information to that available from the existing cancer markers. These early finding now requires validation, initially in retrospective studies but ultimately in large prospective clinical studies. In addition to clinical validation, assays for methylated genes must be robust, simple, standardized, evaluated in external quality assurance schemes and made available at affordable costs. Only then, can patients expect to benefit from measurement of these markers [48].

To interpret the results of the present meta-analysis, some limitations should be taken into account. First, one limitation is that 7 trials had to be excluded from the meta-analysis because they did not provide sufficient data for aggregation. Four out of seven trials detected that *p16* methylation positive related to poor prognosis. However, of the other 3 trials with negative results, two of which carried out the research using relatively larger samples (over 200 patients) still found null association. Moreover, it is known that negative studies are less frequently published or, if they are, with less detailed results, making them less assessable. Although Egger’s test didn’t reach statistical significant, publication bias may influence the results and leads to positive association. Second, eleven out of 18 (61.1%) studies reported HR and corresponding 95%CIs. In order to reduce the missing data, we managed to contact the authors and estimate the outcome with the help of a spreadsheet provided by Tierney *et al* [23]. In spite of the seven outcomes calculated from Kaplan–Meier curve or log-rank test may have introduced some imprecision, we felt this was a risk worth taking in view of the alternative. This situation also highlights the importance of a uniform reporting of study results about survival outcomes. Third, our analysis was based on literatures and not individual patient data. The unavailability of individual patient data could not allow us to correct the potential confounding factors. So, the multivariate analysis cannot be performed, and it cannot be exactly known whether *p16* methylation is a prognostic factor, independently of as known clinical factors, such as age, sex, differentiation, stages, and performance status. Therefore, the results must be interpreted cautiously, because literature-based meta-analysis provides more bias and is less reliable than the individual patient data based analysis [49]. Finally, moderately significant heterogeneity was found in this meta-analysis (I^2^ = 56.7%) for overall survival. Most of the included studies were retrospective and differed in their study designs, such as patient selection, chemotherapeutic protocol and follow-up period. Some trial included patients receiving surgery or radiotherapy in addition to the platinum-based chemotherapy, thus adding the heterogeneity between studies. So we performed the analysis using random-effects model which considers the between-study heterogeneity. Moreover, stratified analyses would be helpful to reduce the heterogeneity and provide additional useful information.

On the basis of the results of this analysis, we support the hypothesis that NSCLC patients with the promoter hypermethylation of *p16* have moderately risk of recurrence and death in all populations considered. Whether p16 methylation testing could move toward routine clinical application as a prognostic tool in NSCLC, it depends on further study validation. Future studies should have a more strict design, with large sample sizes to increase statistical power, a uniform way of analyzing survival outcomes, and a long and specified follow-up period.
